# Supplementary material for: Resource-aware DBSCAN-based re-clustering in hybrid C-V2X/DSRC vehicular networks
Source: PLoS One. 2023 Oct 30;18(10):e0293662. doi: 10.1371/journal.pone.0293662 (PMC10615289; doi:10.1371/journal.pone.0293662)
Supplement: Attachment — Submitted filename: PLOS ONE Clustering.pdf [file pone.0293662.s001.pdf]

Resource- Aware Cluster-based Routing in Hybrid C-V2X/DSRC Vehicular Networks

In this paper, the author proposes Resource aware Cluster-based Routing for C-V2X/DSRC Vehicular communication. The reviewer has several concerns as follows:

1. All abbreviations should be defined in the first place, eg., IEEE, 4G, LTE, 3GPP, UE, RSU, C-V2X..and so on.. vehicle-to-vehicle should be V2V defined in Introduction. In many places abbreviation and acronyms are missed out. The same acronym in abstract and another part of the paper should be defined in abstract and another place in the first place.
2. What are the main challenges in resource-aware cluster based routing for vehicular networks? In order to appeal to interested readers, the authors need to point out the underlying difficulties in the studied work.
3. The study found that few existing algorithms consider negative effects induced by resource management in cellular networks for c-v2x communication. How to evaluate the rationality of the designed criteria considering the kinds of factors of ineffective HO in this study?
4. In this work how to ensure that the quality requirements are satisfied?
5. To provide enough and more information about various communication technologies and brief background about VANET, the following articles are suggested to be used and cited in place of ref 1, 2 in the Introduction.
   <u>New References</u>
   [1] A two-phase fuzzy based access network selection scheme for vehicular ad hoc networks. Peer-to-Peer Networking and Applications. (https://link.springer.com/article/10.1007/s12083-021-01228-w)
   [2] Two-phase access network selection scheme based on weighted sum and game theoretical approaches for vehicular ad hoc networks. Journal of Circuits, Systems and Computers, 30(11), 2150206. (https://www.worldscientific.com/doi/abs/10.1142/S0218126621502066)
   The following article is suggested to be cited instead of reference [11]
   [11]. An efficient data transmission in VANET using clustering method. International Journal of Electronics and Telecommunications. (https://journals.pan.pl/dlibra/show-content?id=106023)
6. Compared with the existing work, what are the key research gaps and contributions of this paper? The authors are suggested to clarify their contribution's specific research problem and novelty in the Introduction Section. Moreover, the reviewed related works in Section II should correspond to the research gaps. Explore the related works in more detail with 3 dimensions: Resource aware, Clustering and Routing. The related work should be re-analysed and reorganised. In related works under the theme routing, I suggest to include the below article instead of Ref. [15]
   [15] A Fuzzy Based Trust And Priority Enabled AODV Routing Scheme For Vehicular Network. INTERNATIONAL JOURNAL OF SCIENTIFIC & TECHNOLOGY RESEARCH.
7. The proposed System model is vague for the reader. Including an illustrative figure for better understanding of the system scenario is highly suggested.
8. The proposed model has 3 variants (3 Algorithms). Why are they necessary? How do they stand during experimental comparison/evaluation?
9. How does the proposed resource-aware cluster based routing approach compare to existing methods in terms of performance and complexity?
10. The authors have well introduced the proposed work, however, the downside of this proposed work is not mentioned in detail. Authors should give a brief explanation related to the disadvantages of the proposed method, and also the main challenges (other than the computational complexity).
11. What do you mean by middle in [16] in Introduction section.

12. Why do you go for DBSCAN clustering mechanism when there are several other mechanisms?
13. Proper references should be provided for the equations that were borrowed from the literature.
14. There are lot of figures without much necessity or explanation in the paper, e.g., Figure 1, 2 which makes the paper less readable. Remove the above figures. The authors are suggested to draw an illustrative figure about clustering in VANET scenario in Introduction section.
15. The comparison between proposed method and traditional method in Figure 5, 6 is insufficient. What is the "traditional method" that is mentioned of? Make comparisons with existing other approaches to validate the results.
16. There remain many typos and grammatical errors. The writing of the paper needs to be improved from informal to formal way. (eg., A question may arise…), please reframe such sentences. Hence it is requested to proofread your text.
