# Supplementary material for: Resource-aware DBSCAN-based re-clustering in hybrid C-V2X/DSRC vehicular networks
Source: PLoS One. 2023 Oct 30;18(10):e0293662. doi: 10.1371/journal.pone.0293662 (PMC10615289; doi:10.1371/journal.pone.0293662)
Supplement: Attachment — Submitted filename: comments plos ONE.docx [file pone.0293662.s002.docx]

**Article ID: PONE-D-23-25645
Title: "Resource-Aware Cluster-based Routing in Hybrid C-V2X/DSRC Vehicular Networks**

Comments:

1. It's interesting to note that DBSCAN, being a well-established clustering algorithm, has been part of the field for a while. The inclusion of re-clustering to form a new methodology adds a fresh perspective to its application. Considering your innovative approach, I wonder if there's room for exploring and producing new clustering concepts within this methodology. This could potentially push the boundaries even further and contribute to the advancement of the field such as grid based , ordering points based ,hierarchy based etc…… It will improve the Quality of ur paper.
2. The article quite informative and appreciate the coverage of the contents. However, it seems that there's a gap in the expansion of certain concepts like DRSC and LTE. Including these would provide a more comprehensive view and enhance the reader's (new) understanding.
3. The references and citations predominantly span up until the year 2015 (avoid 2007). To provide a more up-to-date context and incorporate recent advancements, I recommend considering references beyond that year. This would enhance the relevance and currentness of the article.
4. This article addresses the challenge of unbalanced load. Could you kindly provide more insight into how this is achieved through enhanced DBSCAN approach? Specifically, I'm interested in understanding the separate methodology you've developed to **balance the load within** networks. Elaborating on this aspect would greatly enhance the practical value of the research.
5. the manuscript shows promise in addressing load balancing, clustering, and re-clustering. To enhance clarity, consider adopting a module-wise approach. Clearly outline each module's purpose, methodologies with euations , benefits, and practical cases with suitable diagrams. This will provide readers a systematic understanding. Looking forward to the refined manuscript.
6. The manuscript has highlighted important concerns associated with frequent reclustering, such as resource intensity, overfitting, maintenance challenges, and lack of stability. Addressing these challenges is essential for the practical application of frequent reclustering. Could you elaborate on potential strategies or methodologies that can be employed to overcome or mitigate these drawbacks?
7. In this article, eNodeB is not discussed except in the table. Could you provide a brief explanation of what eNodeB refers to and its importance?
